# Mass gathering events and COVID-19; lessons learnt from the 2020 European football championship 

**DOI:** 10.2471/BLT.23.290044

**Published:** 2024-08-26

**Authors:** Tanja Schmidt, Kazim Beebeejaun, Aimee Latta, Christoph Wippel, Jennifer Addo, Cristiana Salvi, Sarah Tyler, Olha Izhyk, Catherine Smallwood, Ihor Perehinets

**Affiliations:** a WHO Regional Office for Europe, UN City, Marmorvej 51, Copenhagen, DK-2100, Denmark.

## Abstract

Evidence about the impact of mass gatherings during the coronavirus disease 2019 (COVID-19) pandemic on the number of disease cases and on the health-care systems of host countries is limited. Additionally, there have been few publications on the lessons identified from the adaptation of mass gatherings held during the pandemic, including the implementation of comprehensive public health and social measures aimed at reducing viral transmission. This article describes preparations made for the 2020 Union of European Football Associations (UEFA) European Football Championship (UEFA Euro 2020) by the World Health Organization’s (WHO) Regional Office for Europe, UEFA and other stakeholders after the championship had been rescheduled because of the COVID-19 pandemic. Technical guidance on preparations for the football tournament and risk assessment tools were provided by WHO. A task force established by the WHO Regional Office for Europe conducted traditional and event-based disease surveillance before and during UEFA Euro 2020, monitored public health and social measures in the 11 host countries, and developed a risk communication and community engagement strategy that involved multimedia campaigns targeting news and social media, fans, athletes, event organizers and other stakeholders. The lessons and good practices identified during UEFA Euro 2020 are described to help guide preparations for future mass gatherings in health emergencies. Sharing data and recommendations on best practice from previous mass gatherings with the organizers and countries involved in planning for a major event is particularly important.

## Résumé

Peu de preuves existent concernant l'impact des rassemblements de masse lors de la pandémie de maladie à coronavirus 2019 (COVID-19) sur le nombre de cas et sur les systèmes de soins de santé des pays hôtes. En outre, rares sont les publications consacrées aux enseignements qui ont découlé de l'adaptation des rassemblements de masse organisés durant la pandémie, notamment la mise en œuvre de mesures sociales et sanitaires globales visant à limiter la transmission du virus. Le présent article décrit les préparatifs effectués dans le cadre du championnat d'Europe de football 2020 (UEFA Euro 2020) de l'Union européenne des associations de football (UEFA) par le Bureau régional de l'Organisation mondiale de la Santé (OMS) pour l'Europe, l'UEFA et d'autres parties prenantes après le report de la compétition en raison de la pandémie de COVID-19. Des directives techniques relatives à ces préparatifs ainsi que des outils d'évaluation des risques ont été fournis par l'OMS. Un groupe de travail créé par le Bureau régional de l'OMS pour l'Europe a mené une surveillance épidémiologique et événementielle avant et pendant l'UEFA Euro 2020, analysé les mesures sociales et sanitaires dans les 11 pays hôtes et développé une stratégie de communication des risques et d'engagement communautaire impliquant les médias et réseaux sociaux, les supporters, les athlètes, les organisateurs d'événements et d'autres intervenants. Les leçons tirées et les bonnes pratiques identifiées au cours de l'UEFA Euro 2020 sont détaillées ici afin d'aider à encadrer les préparatifs de futurs rassemblements de masse dans des situations d'urgence sanitaire. Partager les données et recommandations sur les meilleures pratiques observées lors de précédents rassemblements de masse avec les organisateurs et pays prenant part à la planification d'un événement de grande envergure est essentiel.

## Resumen

Los datos sobre el impacto de las concentraciones masivas durante la pandemia de la enfermedad por coronavirus de 2019 (COVID-19) en el número de casos de la enfermedad y en los sistemas sanitarios de los países anfitriones son limitados. Además, ha habido pocas publicaciones sobre las lecciones identificadas a partir de la adaptación de las concentraciones masivas celebradas durante la pandemia, incluida la aplicación de medidas sanitarias y sociales integrales destinadas a reducir la transmisión viral. Este artículo describe los preparativos que la Oficina Regional para Europa de la Organización Mundial de la Salud (OMS), la UEFA y otras partes interesadas realizaron para el Campeonato Europeo de Fútbol 2020 de la Unión de Asociaciones Europeas de Fútbol (UEFA) (Eurocopa 2020 de la UEFA) tras la reprogramación del campeonato a causa de la pandemia de la COVID-19. La OMS proporcionó orientación técnica sobre los preparativos del torneo de fútbol y herramientas de evaluación de riesgos. Un grupo de trabajo creado por la Oficina Regional para Europa de la OMS se encargó de la vigilancia de enfermedades tradicional y basada en eventos antes y durante la Eurocopa 2020 de la UEFA, supervisó las medidas sociales y de salud pública en los 11 países anfitriones y elaboró una estrategia de comunicación de riesgos y participación de la comunidad que incluía campañas multimedia dirigidas a los medios de comunicación y las redes sociales, los aficionados, los atletas, los organizadores de eventos y otras partes interesadas. Se describen las lecciones y buenas prácticas identificadas durante la Eurocopa 2020 de la UEFA para ayudar a orientar los preparativos de futuras concentraciones masivas en emergencias sanitarias. Es fundamental compartir los datos y las recomendaciones sobre las mejores prácticas de anteriores concentraciones masivas con los organizadores y los países implicados en la planificación de un evento de gran envergadura.

## ملخص

إن الأدلة محدودة حول تأثير التجمعات الجماهيرية خلال جائحة مرض فيروس كورونا 2019 (كوفيد 19) على عدد حالات الإصابة بالمرض، وعلى أنظمة الرعاية الصحية في الدول المضيفة. بالإضافة إلى ذلك، فقد كان هناك عدد قليل من المنشورات بخصوص الدروس المستفادة من تأقلم التجمعات الجماهيرية التي عقدت أثناء الجائحة، بما في ذلك تنفيذ تدابير الصحة العامة والاجتماعية الشاملة التي تهدف إلى تقييد انتقال الفيروس. تصف هذه المقالة الاستعدادات التي تم إجراؤها لبطولة أمم أوروبا (UEFA Euro 2020)، التابعة للاتحاد الأوروبي لكرة القدم (UEFA)، والتي قام بها المكتب الإقليمي لمنظمة الصحة العالمية (WHO) في أوروبا، والاتحاد الأوروبي لكرة القدم، وأصحاب المصلحة الآخرين بعد إعادة جدولة البطولة بسبب جائحة كوفيد 19. قامت منظمة الصحة العالمية بتقديم إرشادات فنية بخصوص الاستعدادات لبطولة كرة القدم، وأدوات تقييم المخاطر. وقد أجرى فريق عمل، أسسه المكتب الإقليمي لمنظمة الصحة العالمية لأوروبا، مراقبة تقليدية ومبنية على الأحداث قبل وأثناء بطولة أمم أوروبا Euro 2020، وقام الفريق برصد التدابير الصحية والاجتماعية في الدول الإحدى عشر المضيفة، ووضع استراتيجية للتواصل بشأن المخاطر وإشراك المجتمع، تضمنت حملات متعددة الوسائط تستهدف الأخبار، ووسائل التواصل الاجتماعي، والمشجعين، والرياضيين، ومنظمي الفعالية، وغيرهم من أصحاب المصلحة الآخرين. وقد تم وصف الدروس والممارسات الجيدة المستفادة خلال بطولة أمم أوروبا Euro 2020 للمساعدة في توجيه الاستعدادات للتجمعات الجماهيرية المستقبلية في حالات الطوارئ الصحية. إن مشاركة البيانات والتوصيات بخصوص أفضل الممارسات من التجمعات الجماهيرية السابقة، مع الجهات المنظمة والدول المشاركة في التخطيط لحدث كبير، يُعد أمرًا هامًا بشكل خاص.

## 摘要

关于在 2019 冠状病毒病 (COVID-19) 大流行期间大型集会活动对疾病病例数量和东道国卫生保健系统所造成影响的证据有限。此外,介绍在大流行病期间调整举行大型集会活动所获得的相关经验教训(包括实施旨在减少病毒传播的综合性公共卫生和社会措施)的出版物也很少。本文介绍了世界卫生组织 (WHO) 欧洲区域办事处、欧洲足球协会联盟 (UEFA) 和其他利益相关者在确定 2020 年 UEFA 欧洲足球锦标赛 (UEFA Euro 2020) 因 COVID-19 大流行病而改期举办后,为确保其顺利进行所做的准备工作。WHO 为足球锦标赛筹备工作提供了技术指南和风险评估工具。WHO 欧洲区域办事处成立的一个特别工作组在 UEFA Euro 2020 开始之前和进行期间开展了传统和基于事件的疾病监测,监测了 11 个主办国的公共卫生和社会措施实施情况,并制定了风险沟通和社区参与战略,其中包括针对新闻和社交媒体、球迷、运动员、活动组织者和其他利益相关者的多媒体宣传活动。总结了从 UEFA Euro 2020 中所获得的经验教训和良好实践,以帮助指导今后在突发卫生事件情况下举办大型集会活动时如何开展准备工作。与参与重大活动规划的组织者和各国就以往大型集会活动的最佳实践分享相关资料并提供建议尤为重要。

## Резюме

Данные о влиянии массовых мероприятий во время пандемии коронавирусной инфекции 2019 года (COVID-19) на количество случаев заболевания и на системы здравоохранения принимающих стран ограниченны. Кроме того, существует небольшое количество публикаций, посвященных урокам, извлеченным из адаптации массовых мероприятий, проводившихся во время пандемии, включая реализацию комплексных медико-санитарных и социальных мер, направленных на снижение распространения вируса. В этой статье приводится описание подготовки к чемпионату Европы по футболу 2020 года (УЕФА Евро-2020), проведенной Европейским региональным бюро Всемирной организации здравоохранения (ВОЗ), УЕФА и другими заинтересованными сторонами после того, как чемпионат был перенесен из-за пандемии COVID-19. Техническое руководство по подготовке к проведению турнира по футболу и инструменты оценки рисков были предоставлены ВОЗ. Целевой группой, созданной Европейским региональным бюро ВОЗ, проводился традиционный и событийный эпиднадзор за заболеваниями до и во время проведения Евро-2020, мониторинг мер в области здравоохранения и социальной сферы в 11 принимающих странах и разработка стратегии информирования о рисках и вовлечения населения, включающей мультимедийные кампании, направленные на новостные и социальные СМИ, болельщиков, спортсменов, организаторов мероприятий и другие заинтересованные стороны. Приведены уроки и примеры рекомендуемых методов, извлеченные из опыта проведения Евро-2020, которые помогут подготовиться к работе с массовыми скоплениями людей в случае чрезвычайных ситуаций в сфере здравоохранения в будущем. Особенно важен обмен данными и рекомендациями по передовому опыту, полученному в результате ранее проведенных массовых мероприятий, с организаторами и странами, участвующими в планировании крупного события.

## Introduction

Planning and organizing mass gatherings pose public health challenges for organizers, health authorities and governments.^1^^,^^2^ In 2020, the emergence and spread of severe acute respiratory syndrome coronavirus 2 (SARS-CoV-2) intensified these challenges. Mass gatherings were directly affected by the unprecedented global response to the resulting pandemic, and a wide range of public health and social measures were implemented. Moreover, increased attention was paid to mass gatherings as potential drivers of viral transmission in the community and strict restrictions were placed on them. As a result, several events that historically drew large crowds in Europe were cancelled or postponed. Notable examples were the London Marathon, the Pope’s Easter service, the Cannes Film Festival, the Eurovision Song Contest, the Glastonbury Music Festival, the Wimbledon Championships, the Edinburgh Festival and the Munich Oktoberfest.^3^

During the summer of 2021, some countries in the World Health Organization’s (WHO) European Region reported declining trends in coronavirus disease 2019 (COVID-19) cases and deaths, which prompted the resumption of large-scale, in-person events. Although little research has been conducted on the impact of mass gatherings on the COVID-19 pandemic, the 2020 Tokyo Olympic Games were extensively studied and provided valuable insights into mass gathering preparedness during the pandemic. Research found that the Games probably increased COVID-19 cases in Tokyo, and that stringent preventive measures and public health guidance were particularly important.^4^^–^^6^

The decision on whether to restrict, modify, postpone, cancel or proceed with mass gatherings during the COVID-19 pandemic was based on a thorough assessment of all potential public health risks associated with SARS-CoV-2. Preparations for mass gatherings during the pandemic entailed: (i) information-sharing and coordination between stakeholders; (ii) creating event preparedness and response plans specifically tailored to COVID-19; and (iii) informing event attendees about health risks and protective measures.^7^ Precautionary measures adopted during events included establishing surveillance systems to identify symptomatic attendees and modifying the event’s characteristics (e.g. venues, duration or requirements for participation).^7^ After the event ended, coordination between its organizers and local health authorities remained important for information-sharing, epidemiological reporting and contact tracing.^7^

The 2020 European Football Championship organized by the Union of European Football Associations (UEFA), known as UEFA Euro 2020, was originally scheduled to take place in 13 cities in Europe between 12 June and 12 July 2020. However, the tournament was postponed due to the COVID-19 pandemic because of concerns about the health and safety of participating athletes and the potential strain on the health-care systems of countries hosting the football matches.^8^ As part of the rescheduling plan to ensure a safe UEFA Euro 2020 championship, UEFA and host countries partnered with WHO’s Regional Office for Europe to provide technical guidance and support. The aims of this paper are to describe preparations for UEFA Euro 2020 as an illustrative example of mass gathering preparedness during the COVID-19 pandemic, and to report on the lessons learnt and best practices identified to guide future mass gathering preparedness by host countries, event organizers and other stakeholders.

## UEFA Euro 2020 planning and preparation

The World Health Organization (WHO) has a long history of supporting event organizers and host countries in planning and preparing for events involving mass gatherings like UEFA Euro 2020, where there is a risk of infectious disease due to the high density and mobility of participants, including international travel.^9^ Such congregations can lead to close, prolonged and frequent interactions between individuals. Any consequent increase in disease transmission could negatively impact the ability of health systems to respond to emergent crises, both locally and in the home countries to which participants return.^7^^,^^10^

**Fig. 1:**
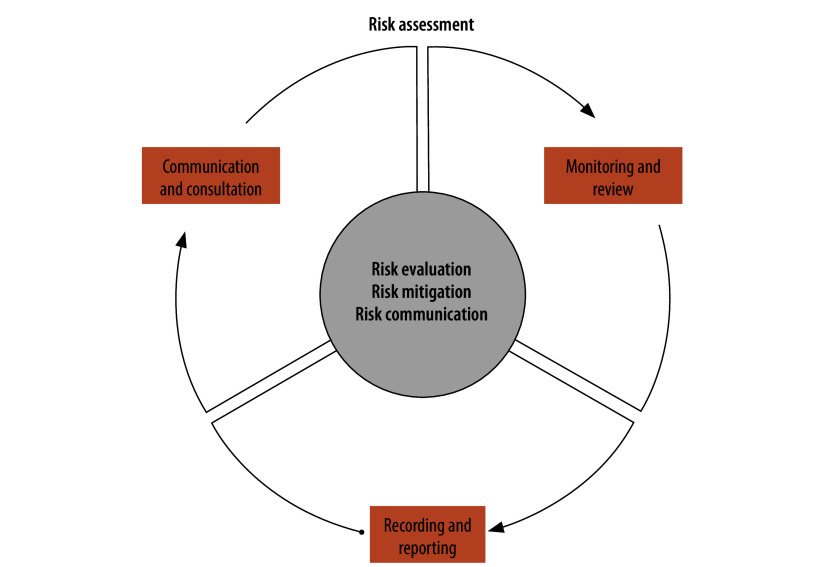
Risk assessment cycle

Source: Adapted from the World Health Organization’s mass gathering coronavirus disease 2019 (COVID-19) risk assessment tool for sports events.^11^

To mitigate the public health risks, WHO recommends that event organizers apply a risk-based approach focused on evaluating, mitigating and communicating risks (Fig. 1).^7^^,^^10^ During the COVID-19 pandemic, risk evaluation involved examining a mass gathering’s characteristics and context to assess the baseline risk of SARS-CoV-2 transmission and the strain on health systems. The communication of risk involved proactively disseminating relevant health information to at-risk populations, and sharing the rationale for precautionary measures in a transparent manner.^7^^,^^10^

### WHO Regional Office for Europe

#### UEFA Euro 2020 task force

In May 2020 before UEFA Euro 2020 took place, the WHO Health Emergencies Programme assembled an internal task force comprising technical specialists from the WHO Regional Office for Europe and personnel from relevant WHO country offices. The task force managed preparedness and response activities (e.g. surveillance, public health and social measures, risk communication and community engagement) and collaborated with public health authorities in WHO European Region Member States. The task force met once a week, or as needed, to discuss the daily monitoring of public health and safety during the football tournament and to respond to public health threats. The task force supported Member States by providing technical guidance, communicating risks, engaging with the community and sharing intelligence. Additionally, it collaborated with the European Centre for Disease Prevention and Control in exchanging daily information on public health threat signals related to the tournament; established an open communication channel with UEFA officials for urgent matters; and remained in direct communication with host countries through national International Health Regulations (2005) focal points.^12^ The task force also documented best practice and lessons learnt for future mass gatherings by publishing research from countries hosting UEFA Euro 2020, and helped update WHO’s risk assessment tools and guidance.^11^^,^^13^^–^^17^

#### Surveillance and measures

The surveillance function of WHO’s internal task force carefully considered WHO’s risk-based approach to planning for mass gatherings, and integrated the approach into every phase of the planning and creation of a COVID-19 surveillance system for UEFA Euro 2020.^7^^,^^10^ The WHO Regional Office for Europe’s approach to continuous public health risk assessment involved employing the key principles of surveillance, risk analysis and reporting.^7^ In combination, two surveillance systems provided timely, useful information for action: traditional surveillance and event-based surveillance. 

Traditional surveillance focused on temporal changes in COVID-19 case rates in each host country (i.e. Azerbaijan, Denmark, Germany, Hungary, Italy, Kingdom of the Netherlands, Romania, the Russian Federation, Spain and United Kingdom of Great Britain and Northern Ireland). The WHO COVID-19 dashboard combined official data from the host countries’ health ministries with findings from laboratories reporting on COVID-19.^18^

In addition, an event-based surveillance system was designed using the Epidemic Intelligence from Open Sources platform, which is a global epidemic intelligence system that detects, processes and curates public health threat signals from open sources, such as news media, social media and official government websites.^19^^,^^20^ Possible public health events can be detected quickly using open source information and, consequently, responses can be faster, thereby helping to prevent excessive morbidity and mortality. For UEFA Euro 2020, public health threat signals related to the tournament were identified using a set of search criteria developed in partnership with the specialized WHO Epidemic Intelligence from Open Sources unit (Box 1). In developing the search criteria, one aim was to balance the sensitivity and specificity with which events of interest could be detected and triage capacity. The goal of the event-based surveillance system was to identify all signals of infectious and noninfectious threats to public health, including chemical, biological, radiological and nuclear threats (Box 1). Surveillance began one month before and continued one month after the tournament (i.e. from 11 May to 11 August 2021).

Box 1 **Examples of keyword search categories for event-based surveillance during UEFA Euro 2020**

**Football celebrations and crowds**

Fanzone; futbol+crowd; football+celebration; fótboli+qeyd+etmɘk.

**Host cities**

Amsterdam; Bucharest; Copenhagen; London; Munich; Seville; St Petersburg.^a^

**Stadiums**

Allianz+Arena; Johan+Cruyff+Arena; Gazprom+Arena;^b^ Olympisch+Stadion+van+Bakoe; Esadio+de+la+Cartuja; Wembley+stadium.

**UEFA Euro 2020**

Championnat+d’Europe+de+football; Labdarúgó-Európa-bajnokság; UEFA+European+Championship; Fuβball-Europameisterschaft.

**Diseases of interest**

COVID-19; diphtheria; mpox; rabies; H1N1 flu.

**Noninfectious threats**

Nerve agents; terrorist attack; sarin; bomb; palladium.

COVID-19: coronavirus disease 2019; UEFA Euro 2020: 2020 Union of the European Football Associations European Football Championship.^a^ search was done using Greek letters.^b^ search was done using Cyrillic letters.Note: The search terms and categories were selected in collaboration with the World Health Organization’s Epidemic Intelligence from Open Sources unit.^20^

Semi-automated, event-based surveillance reports were produced each day. These reports summarized the number of threat signals detected, identified for action and acted upon. An interactive, public-facing surveillance dashboard was produced by the internal WHO task force’s surveillance function: the WHO European Region UEFA Euro 2020 Explorer.^21^ This dashboard, which was shared with WHO European Region Member States, was intended to inform a wide range of audiences (e.g. football fans, the media, governments, public health professionals and researchers) about threat signals detected by WHO surveillance. The dashboard also displayed details of the public health and social measures being implemented. Moreover, information displayed on the dashboard enabled UEFA to accurately assess each host country’s COVID-19 response.^21^

At the onset of the pandemic, the WHO Regional Office for Europe established a systematic method to monitor and analyse the public health and social measures being undertaken in response to COVID-19. Measures implemented by governments in all countries within the WHO European Region were tracked daily, and an index was developed to quantify the severity of the response measures on any given day during the pandemic.^3^^,^^22^

#### Risk communication and community engagement

The WHO Regional Office for Europe developed a series of digital multimedia campaigns that provided tailored, evidence-based information to help reduce public health risks during mass gatherings and to keep spectators, players and communities safe. While acknowledging the delicate balance between protecting people’s health and avoiding socioeconomic damage, the WHO Regional Office for Europe produced regional advice for countries to aid risk-based decision-making the timing, conditions and management of travel and gatherings. In addition, guidance was provided for sports and festival enthusiasts, health authorities and governments through social media channels, including Twitter, Facebook and Instagram.

Public health guidance for UEFA Euro 2020 also involved a broader digital campaign entitled #SummerSense.^23^ In this campaign, a range of messages urged caution while enjoying the warmer months and provided additional advice for people attending the football tournament. There were messages on safe travel,^24^ hand hygiene, physical distancing, mask-wearing and avoiding crowded situations while attending the event.^25^ In addition, messages also addressed the authorities in countries hosting UEFA Euro 2020 and gave guidance on risk-mitigation strategies, such as enhanced testing, contact-tracing and prioritizing vaccination for at-risk groups. To reinforce this advice, the WHO Regional Office for Europe created specific social media content for UEFA Euro 2020, including video clips and fan stories, all of which highlighted the practical implementation of protective measures at the tournament.

There was widespread reporting of these social media messages by the news media. Sports news providers such as BBC Sport and international news agencies (e.g. AFP, Reuters and Associated Press News) featured interviews with WHO technical spokespeople, and a series of question-and-answer items were disseminated across websites and social media platforms in Europe.^26^ In addition, WHO representatives in several host countries successfully persuaded newspapers to place articles on measures to prevent viral transmission opposite the editorial page.^27^ Overall, in June and July during UEFA Euro 2020, the WHO Regional Office for Europe was responsible for generating over 2000 media articles that reached an estimated audience of up to 7 billion people (estimates from Signal AI, London, United Kingdom of Great Britain and Northern Ireland, in September 2021).

### UEFA

In developing a common approach to mitigating the risk of disease spread, UEFA produced a recovery strategy and proposed a timeline for coordinating COVID-19 public health measures between local authorities and UEFA. A separate COVID-19 task force was embedded within UEFA’s medical committee. The function of this task force, which comprised medical professionals, was to coordinate and align preventive measures among health authorities in countries hosting UEFA Euro 2020. As part of UEFA’s risk assessment, three roundtable discussions involving participants from host countries’ health authorities were organized before the tournament. Topics for discussion included the evolution of health measures in host cities and countries that were implementing recovery plans, and the role that football authorities could play in supporting these plans. In addition, WHO’s risk-assessment tool for mass gatherings was adapted to ensure that the specific public health and social measures recommended for the tournament were implemented in host countries and that health authorities were involved in the process.^11^ Various scenarios were appraised by UEFA in partnership with host countries, including the risk associated with different stadium capacities for matches and fan zones, given the local COVID-19 case rate. Local health authorities made the final decision on which measures to implement.

In collaboration with host countries, UEFA requested that a minimum level of preventive measures were implemented to minimize disease spread. For example, in addition to strict mask policies, UEFA proposed strict guidance on the channelling and flow of spectators. Also, UEFA implemented a COVID-19 spectator communications strategy to provide fans with health guidance, including on how to make an informed decision about protecting their health, and to publicize the public health and social measures currently in place in stadiums. Information was disseminated through digital platforms, such as mobile phone applications, emails and social media posts. Push notifications were available at stadium entry for fans who opted in to receiving reminders of public health and social measures, including daily tips to how to keep safe. Physical information channels included signage, stadium screens, spectator information points and verbal advice from volunteers.

The communication strategy developed by UEFA was based on operational documents, including UEFA’s return-to-play protocol, and minimum health and hygiene requirements for the return of spectators.^28^^,^^29^ The strategy also drew on recommendations from external sources, including: (i) guidance provided by WHO;^27^ (ii) guidance from the European Club Association (ECA); and (iii) guidance from the *Fédération Internationale de Football Association* (FIFA).^30^

### Individual countries

#### Trends in COVID-19 cases

**Fig. 2:**
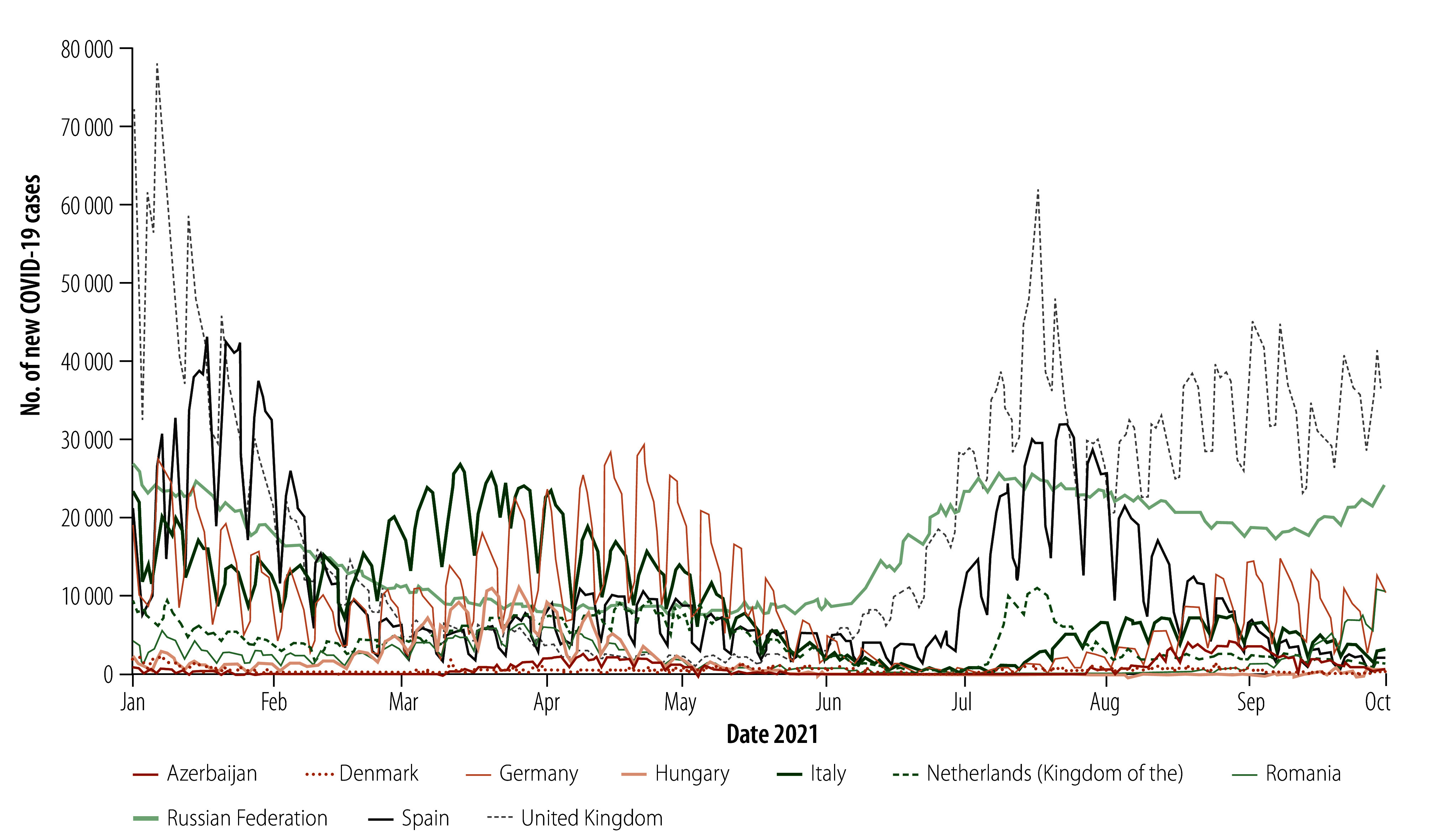
New COVID-19 cases in countries hosting UEFA Euro 2020, January to October 2021

Close to the start of the tournament, COVID-19 case numbers were increasing in the Russian Federation and the United Kingdom and there was a continued, marked rise throughout the championship. Although other countries initially reported decreasing case numbers, the trend changed and an uptick in new cases was observed across all host countries during the tournament (Fig. 2).

COVID-19: coronavirus disease 2019; UEFA Euro 2020: 2020 Union of the European Football Associations European Football Championship.

Notes: Case numbers are presented for the United Kingdom of Great Britain and Northern Ireland as a whole rather than for England and Scotland separately. The football tournament took place between 11 June and 11 July 2021.

#### Public health and social measures

**Fig. 3:**
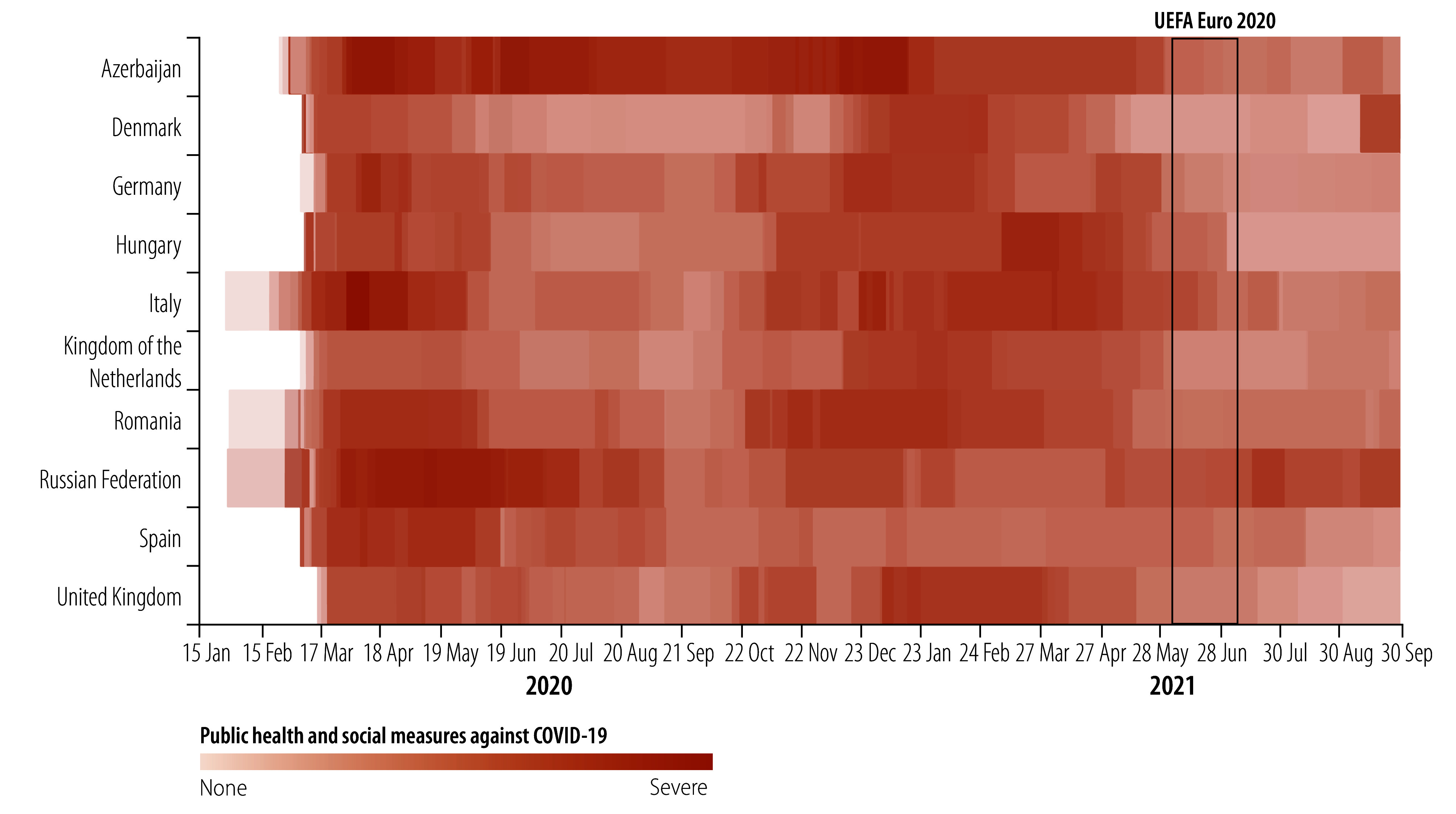
Severity of public health and social measures against COVID-19 in countries hosting UEFA Euro 2020, January 2020 to September 2021

In several stadiums, the public health and social measures adopted differed from those of the host countries (Table 1). For example, mask policies were stricter or quotas for gatherings were lower than otherwise allowed. Generally, host countries were in the process of easing restrictions in the lead up to, and during, the tournament (Fig. 3). One exception was the Russian Federation, where public health and social measures were being strengthened locally in Moscow and Saint Petersburg in response to a rise in COVID-19 cases in early to mid-June. At the beginning of the tournament, mask mandates were still active in all host countries. In Italy, Romania and Spain, people were required to wear masks in all outdoor and indoor public spaces. In Azerbaijan, Germany, Kingdom of the Netherlands, the Russian Federation and the United Kingdom, mask-wearing was mandated in certain indoor public spaces. Azerbaijan eased the requirement for masks outdoors one month before the tournament, and Italy lifted its general indoor and outdoor mask mandate at the end of June 2021. The mandated form of face-covering in the German federal state of Bavaria, where the host stadium was located, was an FFP2, KN95 or equivalent mask. A few weeks before the tournament started, Hungary required masks only on public transportation. All businesses, including hospitality businesses, were open in Azerbaijan, Denmark, Germany, Hungary, Italy, Kingdom of the Netherlands, the Russian Federation, Spain and United Kingdom; in the Kingdom of the Netherlands, customers had to present a so-called COVID-19 pass as proof of immunity or of a negative test result. England had entered the third stage of its roadmap for exiting lockdown, during which all indoor hospitality venues, including pubs, could reopen to the public. Although restrictions on gatherings varied widely between host countries, a distinction was typically made between private and public gatherings.

**Table 1:** Public health and social measures against COVID-19 in stadiums and generally during the 2020 UEFA European Football Championship, 11 June to 11 July 2021

Stadium	City, country	Capacity allowed, %	No. spectators allowed (full capacity)	Public health and social measures in stadium	National or subnational public health and social measures
Johan Cruijff Stadium	Amsterdam, Netherlands (Kingdom of the)	22	12 000 (54 000)	(i) Physical distancing: 1.5 m; (ii) masks: always, except when seated; and (iii) stadium entry: timed slots and only with a negative COVID-19 test result	(i) Masks: required only on public transport; (ii) schools: open; (iii) businesses: all, including hospitality businesses, open to customers with a COVID-19 pass; and (iv) gatherings: event venues at two-thirds capacity
Baku Olympic Stadium	Baku, Azerbaijan	50	34 500 (69 000)	(i) Physical distancing: 1.5 m; (ii) masks: always; and (iii) stadium entry: timed slots with no negative COVID-19 test result or proof of vaccination required	(i) masks: requirement recently lifted for outdoor spaces; (ii) schools: generally open but could be closed subnationally depending on the epidemiological situation; (iii) businesses: all open, including hospitality businesses; and (iv) gatherings: public gatherings limited to 150 participants
National Arena Bucharest	Bucharest, Romania	24	13 000 (54 000)	(i) Physical distancing: 1.5 m; (ii) masks: always, except when seated; and (iii) stadium entry: timed slots and only with a negative COVID-19 test result	(i) Masks: required in all public indoor and outdoor spaces; (ii) schools: partially closed in regions with a high incidence of infection; (iii) businesses: mostly open but hospitality businesses closed in regions with a high incidence; and (iv) gatherings: social gatherings limited to small groups
Puskás Aréna	Budapest, Hungary	100	68 000 (68 000)	(i) Physical distancing: 1.5 m; (ii) masks: recommended; and (iii) stadium entry: timed slots and temperature checks	(i) Masks: required only on public transport; (ii) schools: open; (iii) businesses: all open, including hospitality businesses; and (iv) gatherings: events limited to 500 participants but special sports events exempt from this cap
Parken Stadium	Copenhagen, Denmark	48	15 900 (33 000)	(i) Physical distancing: unspecified; (ii) masks: always, except when seated; and (iii) stadium entry: timed slots and only with a negative COVID-19 test result or proof of vaccination	(i) Masks: required on public transport; (ii) schools: open; (iii) businesses: all open, including hospitality businesses; and (iv) gatherings: maximum 5000 individuals at outdoor events and 250 at indoor events
Hampden Park	Glasgow, United Kingdom	23	12 000 (51 866)	(i) Physical distancing: 1.5 m; (ii) masks: always, except when seated; and (iii) stadium entry: timed slots with no negative COVID-19 test result or proof of vaccination required	(i) Masks: required on public transport; (ii) schools: open; (iii) businesses: all open, including hospitality businesses; and (iv) gatherings: indoor events limited to 100 individuals and outdoor events limited to 1000
Wembley	London, United Kingdom	25	22 500 (90 000)	(i) Physical distancing: unspecified; (ii) masks: always, except when seated and looking forward; and (iii) stadium entry: timed slots and only with a negative COVID-19 test result or proof of vaccination	(i) Masks: required on public transport; (ii) schools: open; (iii) businesses: all open, including hospitality businesses; and (iv) gatherings: events with up to 30 people allowed
Football Arena Munich	Munich, Germany	21	14 500 (70 000)	(i) Physical distancing: 1.5 m; (ii) masks: always, with FFP2 mask required; and (iii) stadium entry: timed slots and only with a negative COVID-19 test result or proof of vaccination	(i) Masks: FFP2 masks required in Bavaria in all public indoor spaces and on public transport; (ii) schools: open; (iii) businesses: all open, including hospitality businesses; and (iv) gatherings: events with ≤ 50 people indoors and ≤ 100 people outdoors allowed
Stadio Olimpico	Rome, Italy	23	16 000 (68 530)	(i) Physical distancing: 1 m; (ii) masks: always; and (iii) stadium entry: timed slots and only with a negative COVID-19 test result or proof of vaccination	(i) Masks: general indoor and outdoor requirement lifted on 28 June 2021 and, thereafter, masks were required only for large gatherings; (ii) schools: open; (iii) businesses: all open, including the hospitality sector; and (iv) gatherings: no restrictions
Saint Petersburg Stadium	Saint Petersburg, Russian Federation	50	30 500 (61 000)	(i) Physical distancing: no requirement; (ii) masks: always; and (iii) stadium entry: timed slots but no negative COVID-19 test result or proof of vaccination required	(i) Masks: required in all indoor public spaces; (ii) schools: open; (iii) businesses: all open including hospitality businesses; and (iv) gatherings: events limited to 3000 people
Stadium La Cartuia Sevilla	Seville, Spain	30	18 000 (60 000)	(i) Physical distancing: 1.5 m; (ii) masks: always; (iii) stadium entry: timed slots but no negative COVID-19 test result or proof of vaccination required	(i) Masks: required in indoor and outdoor public spaces in Andalusia; (ii) schools: open; (iii) businesses: all open at 75% capacity, including hospitality businesses; and (iv) gatherings: no limitations

COVID-19: coronavirus disease 2019; FFP: filtering face piece; UEFA: Union of the European Football Associations.

COVID-19: coronavirus disease 2019; UEFA Euro 2020: 2020 Union of the European Football Associations European Football Championship.

Notes: The severity of public health and social measures is presented for the United Kingdom of Great Britain and Northern Ireland as a whole rather than for England and Scotland separately. The football tournament took place between 11 June and 11 July 2021.

#### International travel

In response to the spread of the delta variant (B.1.617.2) of SARS-CoV-2, the countries of seven of the 11 cities hosting UEFA Euro 2020 introduced an entry ban, testing requirements or quarantine measures affecting access to the country before the championship began.^3^ The other four host countries adjusted their restrictions on international travel specifically to allow attendance at the matches. Azerbaijan, for example, lifted entry restrictions for international spectators who had an official invitation from UEFA or a ticket for a match in Baku; only a predeparture negative polymerase chain reaction (PCR) test result for SARS-CoV-2 was required. Hungary announced that foreign attendees could enter the country with a negative PCR test result, but could only access accommodation and catering and leisure facilities with a valid match ticket. Germany announced that people who were involved in preparations for, or who participated in, the event were exempt from quarantine requirements, even if they had travelled from an area where there was a virus variant of concern. This exemption did not include fans, who were still obliged to follow the government’s self-isolation rules.

#### Country surveillance systems for UEFA Euro 2020

Several host countries implemented enhanced surveillance during the tournament, including Denmark, Germany, Italy and the United Kingdom.^13^^–^^17^ A combination of event-based, traditional and contact-based surveillance was used to assess the impact of the tournament on SARS-CoV-2 transmission. Public Health Scotland, for example, used data from a contact-tracing surveillance system to link attendance at events to positive laboratory test results.^13^

## Discussion

Several lessons can be learnt from the actions taken by the WHO Regional Office for Europe and UEFA in preparation for UEFA Euro 2020. The establishment of a task force, which included relevant stakeholders, by the WHO Regional Office for Europe early in the planning stage laid the foundations for effective coordination between UEFA, WHO Regional Office for Europe technical units and host countries both before and during the tournament. The task force functioned as a conduit for sharing information, technical guidance and best practice that was vital for assessing the potential impact of holding UEFA Euro 2020 during the COVID-19 pandemic, and for guiding decision-making and preparedness and mitigation measures. Before, during and after UEFA Euro 2020, the key decision-makers on which public health and social measures should be implemented were the governments and health authorities of the host countries and of other European countries from which large numbers of spectators travelled. The WHO Regional Office for Europe supported decision-making by providing countries with the latest surveillance data, informing them about changes to public health and social measures, and distributing health information and advice targeted to participants. In health emergencies, good coordination between stakeholders is essential for ensuring that risk assessments and mitigation efforts are robust, for preventing disease spread and for minimizing the strain on national health systems.^7^

Coordinating support activities in a rapidly changing and uncertain environment was challenging. For example, it was difficult to document and analyse up-to-date information on the public health and social measures being undertaken because host cities adopted different approaches, and because changes were continuously being made before and during the tournament. Establishing a dedicated monitoring team, using a standard classification for public health and social measures, and providing regular updates to the task force were crucial for overcoming these obstacles. Tailoring risk communication and community engagement activities to UEFA Euro 2020 was also challenging because such occasions attract a diverse audience with different perceptions of risk and with a varying degree of willingness to comply with protective measures. Regular and rapidly adaptive social listening and message testing were vital for overcoming these challenges and for providing the target audience with appropriate public health information and advice.

The COVID-19 surveillance system designed for UEFA Euro 2020 was useful for both planning and preparation, and provided valuable information to the task force. Although the WHO Regional Office for Europe’s surveillance dashboard was not formally evaluated, the public availability of epidemiological and surveillance data and of information on public health and social measures across host countries in real time was a vital resource for UEFA Euro 2020 decision-makers confronting one of the first multicity mass gatherings to have taken place during a global pandemic. A key gap identified, which should be considered for future mass gatherings, was the lack of a robust framework for evaluating surveillance systems for mass gatherings using quantitative and qualitative data.

Another lesson from UEFA Euro 2020 was that public health and social measures had to be extended beyond event sites to include areas directly outside stadiums, such as fan zones and pubs. This extension was crucial because large crowds tended to congregate in these peripheral areas, which created opportunities for viral transmission that could have undermined the measures taken inside the main event zones. Ensuring that public health measures are implemented in all areas where fans gather helps mitigate the risk of community transmission. Future event planning, therefore, needs to consider gatherings related to events and not only those at event sites.^7^^,^^10^

Communicating information on health risks to event attendees and the broader host community is also a crucial part of event planning. Mass gatherings offer a powerful opportunity to reach individuals with important health messages, especially those who may not typically exhibit proactive, health-seeking behaviour. Disseminating WHO public health guidance during UEFA Euro 2020 raised public awareness because health advice was provided through accessible media platforms and via familiar messengers such as players and fans. Repeatedly, global emergencies underscore the importance of conveying messages through trusted channels of communication.^7^

## Conclusion

Our description of the preparedness activities undertaken by different stakeholders for UEFA Euro 2020 during the COVID-19 pandemic adds to the limited evidence available on organizing public health activities during mass gatherings. Our experience shows that sharing data and recommendations on best practice from previous mass gatherings with the organizers and countries involved in planning for a major event is crucial for: (i) refining contact-tracing; (ii) slowing the spread of infectious disease; and (iii) understanding the impact of mass gatherings during an evolving public health emergency.

## References

[1] Tavan A, Tafti AD, Nekoie-Moghadam M, Ehrampoush M, Vafaei Nasab MR, Tavangar H, et al. Risks threatening the health of people participating in mass gatherings: a systematic review. J Educ Health Promot. 2019 Oct 24;8(1):209. 10.4103/jehp.jehp_214_19 31807599 PMC6852309

[2] Karami M, Doosti-Irani A, Ardalan A, Gohari-Ensaf F, Berangi Z, Massad E, et al. Public health threats in mass gatherings: a systematic review. Disaster Med Public Health Prep. 2019 Dec;13(5-6):1035–46. 10.1017/dmp.2018.161 31250774

[3] Public health and social measures in response to COVID-19 [internet]. Copenhagen: WHO Regional Office for Europe: 2024. Available from: https://phsm.euro.who.int/covid-19 [cited 2024 Feb 28].

[4] Yoneoka D, Eguchi A, Fukumoto K, Kawashima T, Tanoue Y, Tabuchi T, et al. Effect of the Tokyo 2020 Summer Olympic Games on COVID-19 incidence in Japan: a synthetic control approach. BMJ Open. 2022 Sep 20;12(9):e061444. 10.1136/bmjopen-2022-061444 36127076 PMC9490294

[5] Yamamoto N, Mitsuhashi T, Tsuchihashi Y, Yorifuji T. Causal effect of the Tokyo 2020 Olympic and Paralympic Games on the number of COVID-19 cases under COVID-19 pandemic: an ecological study using the synthetic control method. J Pers Med. 2022 Feb 3;12(2):209. 10.3390/jpm12020209 35207697 PMC8879008

[6] Gabrielli AF, Glaria AA, Borodina M, Mullen L, Watson CR, Kobokovich A, et al. Risk-based management of international sporting events during the COVID-19 pandemic. Bull World Health Organ. 2024 Aug 1;102(8):608–14. 10.2471/BLT.23.290034 39070599 PMC11276152

[7] Key planning recommendations for mass gatherings in the context of COVID-19: interim guidance, 29 May 2020. Geneva: World Health Organization; 2020. Available from: https://iris.who.int/handle/10665/332235 [cited 2024 Feb 28].

[8] UEFA postpones EURO 2020 by 12 months. Nyon: Union of European Football Associations; 2020. Available from: https://www.uefa.com/news-media/news/025b-0f8e76aef315-8506a9de10aa-1000--uefa-postpones-euro-2020-by-12-months/ [cited 2024 Feb 28].

[9] Smallwood CAH, Arbuthnott KG, Banczak-Mysiak B, Borodina M, Coutinho AP, Payne-Hallström L, et al. Euro 2012 European Football Championship finals: planning for a health legacy. Lancet. 2014 Jun 14;383(9934):2090–7. 10.1016/S0140-6736(13)62384-3 24857705

[10] Holding gatherings during the COVID-19 pandemic: WHO policy brief, 2 August 2021. Geneva: World Health Organization; 2021. Available from: https://www.who.int/publications/i/item/holding-gatherings-during-the-covid-19-pandemic-who-policy-brief-2-august-2021 [cited 2024 Feb 28].

[11] WHO mass gathering COVID-19 risk assessment tool – sports events. Guidance for authorities and organizers of sports events planning mass gatherings during the current COVID-19 pandemic. Geneva: World Health Organization; 2020. Available from: https://www.who.int/publications/i/item/10665-333187 [cited 2024 Jul 26].

[12] National IHR focal points. Geneva: World Health Organization; 2024. Available from: https://www.who.int/teams/ihr/national-focal-points#:~:text=The%20Regulations%20define%20a%20National,Contact%20Points%20under%20these%20Regulations%22 [cited 2024 Jul 25].

[13] Marsh K, Griffiths E, Young JJ, Gibb C-A, McMenamin J. Contributions of the EURO 2020 football championship events to a third wave of SARS-CoV-2 in Scotland, 11 June to 7 July 2021. Euro Surveill. 2021 Aug;26(31):2100707. 10.2807/1560-7917.ES.2021.26.31.2100707 34355691 PMC8343549

[14] Smith JAE, Hopkins S, Turner C, Dack K, Trelfa A, Peh J, et al. Public health impact of mass sporting and cultural events in a rising COVID-19 prevalence in England. Epidemiol Infect. 2022 Jan 31;150:e42. 10.1017/S0950268822000188 35094727 PMC9058658

[15] Heese H, Marquis A, Diercke M, Markus I, Böhm S, Metz J, et al. Results of the enhanced COVID-19 surveillance during UEFA EURO 2020 in Germany. Epidemiol Infect. 2022 Mar 3;150:1–18. 10.1017/S0950268822000449 35236530 PMC8924559

[16] Bennedbæk M, Button MSF, Nielsen LB, Bybjerg-Grauholm J, Wiid Svarrer C, Møller KL, et al. Increased transmission of SARS-CoV-2 in Denmark during UEFA European championships. Epidemiol Infect. 2022 Mar 23;150:e123. 10.1017/S095026882200019X 35317884 PMC9254153

[17] Riccardo F, Frisicale EM, Guzzetta G, Ferraro F, Merler S, Maringhini G, et al. Winning during a pandemic: epidemiology of SARS-CoV-2 during EURO2020 in Italy. Epidemiol Infect. 2022 Apr 22;150:e166. 10.1017/S0950268822000723 35450542 PMC9509794

[18] WHO. COVID-19 dashboard [internet]. Geneva: World Health Organization; 2024. Available from: https://data.who.int/dashboards/covid19/cases?n=c [cited 2024 Feb 28].

[19] Abdelmalik P, Peron E, Schnitzler J, Fontaine J, Elfenkämper E, Barboza P. The Epidemic Intelligence from Open Sources initiative: a collaboration to harmonize and standardize early detection and epidemic intelligence among public health organizations. Wkly Epidemiol Rec. 2018;93:267–8.

[20] Epidemic Intelligence from Open Sources. Zero impact from health threats. Geneva: World Health Organization; 2024. Available from: https://www.who.int/initiatives/eios [cited 2024 Jul 25].

[21] WHO European Region UEFA EURO 2020 explorer [internet]. Copenhagen: WHO Regional Office for Europe: 2024. Available from: https://who.maps.arcgis.com/apps/dashboards/2e328f146c34408d808bba6ea6d18331 [cited 2024 Feb 28].

[22] A systematic approach to monitoring and analysing public health and social measures (PHSM) in the context of the COVID-19 pandemic: underlying methodology and application of the PHSM database and PHSM Severity Index. Copenhagen: WHO Regional Office for Europe: 2020. Available from: https://iris.who.int/handle/10665/337686 [cited 2024 Feb 28].

[23] With the pandemic far from over, we all need to practice #SummerSense. Geneva: World Health Organization; 2021. Available from: https://www.who.int/europe/news/item/10-06-2021-with-the-pandemic-far-from-over-we-all-need-to-practice-summersense [cited 2024 Jul 25].

[24] World Health Organization Regional Office for Europe. With the #COVID19 pandemic far from over, we all need to practice #SummerSense [tweet 2021 Jun 26]. X Corp: San Francisco; 2021. Available from: https://x.com/WHO_Europe/status/1408703335306309632 [cited 2024 Feb 28].

[25] World Health Organization Regional Office for Europe. The #EURO2020 may be nearly over, but stay safe while enjoying the football. We all need to practice #SummerSense [tweet 2021 Jul 9]. X Corp: San Francisco; 2021. Available from: https://x.com/WHO_Europe/status/1413429971897307140 [cited 2024 Feb 28].

[26] Our actions will determine end of pandemic: WHO officials. UN health agency representatives urge world to get vaccinated, adhere to safety measures in summer. Ankara: Anadolu Ajansı; 2021. Available from: https://www.aa.com.tr/en/health/our-actions-will-determine-end-of-pandemic-who-officials/2306848 [cited 2024 Feb 28].

[27] Considerations for sports federations/sports event organizers when planning mass gatherings in the context of COVID-19: interim guidance. Geneva: World Health Organization; 2020. Available from: https://www.who.int/publications-detail-redirect/considerations-for-sports-federations-sports-event-organizers-when-planning-mass-gatherings-in-the-context-of-covid-19-interim-guidance [cited 2024 Feb 28].

[28] The UEFA return to play protocol. Basel: Union of European Football Associations; 2021. Available from: https://www.uefa.com/news-media/news/0265-114289c53314-947427af4d25-1000--the-uefa-return-to-play-protocol/ [cited 2024 Jul 25].

[29] UEFA minimum health and hygiene requirements for the return of spectators. Basel: Union of European Football Associations; 2020. Available from: https://editorial.uefa.com/resources/0262-1081a4df2c6d-98022dcc8f82-1000/uefa_minimum_health_hygiene_requirements_for_the_return_of_spectators_.pdf [cited 2024 Jul 26].

[30] Guidance on return to play with fans. Nyon: European Club Association; 2020. Available from: https://www.ecaeurope.com/media/4799/20200825_guidance_return-to-play-with-fans.pdf [cited 2024 Feb 28].

